# Bio-Inspired Energy-Efficient Routing for Wireless Sensor Networks Based on Honeybee Foraging Behavior and MDP-Driven Adaptive Scheduling

**DOI:** 10.3390/biomimetics11050311

**Published:** 2026-05-01

**Authors:** Fangyan Chen, Xiangcheng Wu, Weimin Qi, Zhiming Wang, Zhiyu Wang, Peng Li

**Affiliations:** 1 School of Artificial Intelligence, Jianghan University, Wuhan 430056, China; fychen@stu.jhun.edu.cn (F.C.); leolink@jhun.edu.cn (Z.W.); zywang@stu.jhun.edu.cn (Z.W.); 2 School of Intelligent Manufacturing, Jianghan University, Wuhan 430056, China; lpeng520@jhun.edu.cn

**Keywords:** bio-inspired routing, honeybee foraging, wireless sensor networks, mixed-integer linear programming, Q-learning, adaptive trajectory planning, energy efficiency

## Abstract

Wireless Sensor Networks (WSNs) enable energy-efficient data collection in dynamic environments but continue to face the dual challenges of severely constrained node energy and the spatiotemporal heterogeneity of data traffic. Inspired by honeybee foraging behavior, this paper proposes a hybrid optimization framework that integrates mixed-integer linear programming (MILP) and Markov decision processes (MDP), utilizing Q-learning for adaptive decision-making. The proposed framework systematically maps the dual-layer decision-making mechanism of honeybee foraging onto a synergistic architecture combining MILP-based global planning and MDP-based local adaptation, offering a novel bio-inspired solution for mobile sink trajectory planning and adaptive routing. Specifically, the upper-level MILP module simulates a colony-level global assessment of distant nectar sources, generating an initial global trajectory by determining the optimal access sequence of cluster heads to minimize the movement cost of the mobile sink. The lower-level Q-learning module simulates the individual-level local adaptation, where bees adjust harvesting behavior in real-time based on nectar quality and distance. This module continuously optimizes routing parameters based on real-time network states, including residual energy, the ratio of surviving nodes, data queue lengths, and cluster head density. The algorithm employs an ϵ-greedy strategy to balance exploration and exploitation, while a periodic decision-update mechanism is introduced to harmonize computational efficiency with learning stability. Furthermore, a multi-objective reward function is designed to jointly optimize energy efficiency, network lifetime, end-to-end latency, and path length. Extensive simulation results demonstrate that the proposed MILP-MDP hybrid framework significantly outperforms several representative baseline algorithms in terms of network lifetime extension and energy balance. These findings validate that the integration of bio-inspired foraging strategies and reinforcement learning provides an efficient and robust solution for trajectory planning and adaptive routing in dynamic WSNs.

## 1. Introduction

With the rapid advancement of the Internet of Things (IoT), wireless sensor networks (WSNs) have become a fundamental infrastructure for intelligent sensing systems across a wide range of applications, including environmental monitoring, smart manufacturing, military surveillance, and urban sensing. A typical WSN consists of a large number of low-power sensor nodes that cooperatively perform sensing and data transmission tasks through self-organized communication. However, due to limited battery capacity, constrained communication range, and redundant data generation, achieving efficient energy utilization while maintaining reliable data collection remains a fundamental challenge in WSN design.

One of the most critical issues in traditional WSN architectures is the imbalance in energy consumption among nodes. In networks with a static sink, nodes located close to the sink must relay large volumes of data generated by distant nodes, leading to significantly faster energy depletion compared to other nodes. This phenomenon leads to the well-known “energy hole” problem. Once such nodes exhaust their energy, network connectivity may be disrupted even though many other nodes still possess sufficient residual energy. Therefore, balancing energy consumption across the network while maintaining communication efficiency is essential for prolonging the network lifetime.

To mitigate the energy hole problem, the use of mobile sinks has attracted extensive attention in recent years. By periodically changing the position of the sink, the communication load can be redistributed among different regions of the network, thereby balancing energy consumption and extending the overall network lifetime. However, introducing sink mobility also brings new optimization challenges. Specifically, the trajectory planning and dwell scheduling of a mobile sink must simultaneously consider energy consumption, communication delay, and network coverage. An excessively long trajectory may increase data collection latency, while frequent sink repositioning may introduce additional energy overhead and scheduling complexity. Consequently, achieving an effective trade-off between energy efficiency and communication delay remains a key research problem in mobile sink WSNs.

Despite these advances, most existing studies treat mobile sink trajectory planning and routing adaptation as loosely coupled or sequential optimization problems. This separation limits the ability to jointly optimize long-term energy balance and short-term network dynamics under time-varying conditions.

More importantly, even in existing hybrid or adaptive frameworks, the interaction between global trajectory decisions and local routing policies is typically indirect or delayed. For example, trajectory planning is often performed offline or updated at coarse time scales, while routing adaptation reacts to instantaneous network states without explicit feedback to the global planner. This lack of bidirectional coupling prevents the system from achieving consistent system-level optimality under dynamic conditions.

Therefore, a more precise unresolved problem can be formulated as follows: that of how to design a unified optimization-learning architecture in which (i) global trajectory planning is aware of evolving network states, and (ii) local routing decisions are constrained by globally optimized structural policies, while maintaining computational tractability.

Moreover, while reinforcement learning (RL) and hybrid optimization methods have been introduced, they often lack a principled mechanism to integrate global deterministic optimization with local adaptive decision-making in a unified framework.

### 1.1. Honeybee Foraging Behavior: A Bionic Paradigm for Mobile Sink WSNs

Nature-inspired computing has emerged as a powerful paradigm for solving complex optimization problems in decentralized and dynamic systems. Among various biological systems, the foraging behavior of honeybee (*Apis mellifera*) colonies demonstrates remarkable collective intelligence and adaptive efficiency, honed through millions of years of evolution. This behavior provides an ideal bionic model for addressing the global–local optimization challenges in mobile sink-based wireless sensor networks (WSNs).

Honeybee foraging operates through a sophisticated dual-phase mechanism with a clear division of labor:

(1) Scout bees perform global exploration, identifying promising food sources and sharing information at the colony level.
(2) Forager bees perform local exploitation, dynamically adjusting their behavior based on environmental feedback.

Unlike conventional bio-inspired algorithms that mainly provide heuristic search strategies, the honeybee paradigm here is used as a structural design principle. Specifically, it enforces a hierarchical separation between global optimization and local adaptation, while simultaneously requiring continuous information exchange between the two layers. This property directly motivates the integration of MILP and MDP within a unified framework.

**Figure 1:**
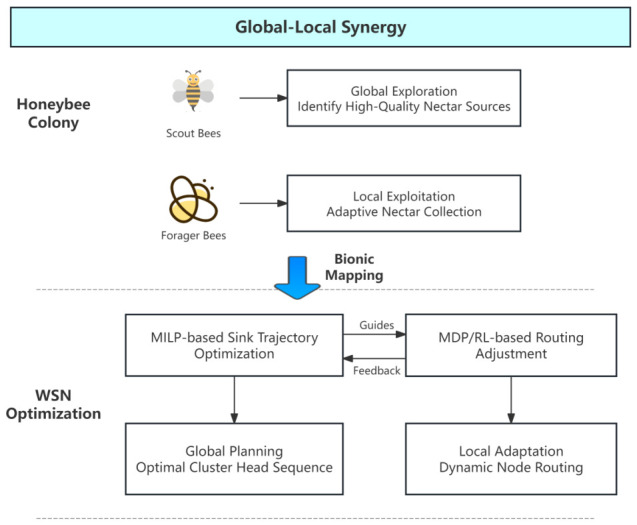
Bionic mapping of honeybee foraging behavior to the MILP-MDP framework for mobile sink WSNs.

As illustrated in Figure 1, the elegant “global exploration + local adaptation” synergy in honeybee colonies offers a precise biological analog for mobile sink WSNs. The global assessment performed by scout bees naturally maps to the **Mixed-Integer Linear Programming (MILP)** module, which determines the optimal visiting sequence and dwell times of the mobile sink at cluster heads to minimize total movement cost and energy consumption. Meanwhile, the adaptive behavior of forager bees corresponds to the **Markov Decision Process (MDP)** and reinforcement learning (RL) framework, enabling individual sensor nodes to dynamically adjust routing decisions based on real-time network states.

Unlike conventional bio-inspired approaches that primarily serve as heuristic analogies, the proposed mapping establishes an explicit correspondence between biological roles and algorithmic components, enabling a structured integration of optimization and learning.

Furthermore, this mapping is not only conceptual but also operational: the MILP layer produces structured policies (e.g., visiting order and dwell constraints) that define the feasible action space for the MDP layer, while the MDP layer provides state-dependent feedback that can trigger re-optimization in the MILP layer.

By integrating these two hierarchical layers, the proposed framework achieves a synergistic global–local optimization architecture: the MILP layer provides long-term strategic trajectory planning, while the MDP/RL layer offers short-term adaptive responsiveness. This bio-inspired design effectively balances energy efficiency and communication latency in dynamic WSN environments.

### 1.2. Related Work

Existing literature has tackled WSN energy optimization through three primary technological trajectories: heuristic mechanisms, metaheuristic optimization, and intelligent learning. These approaches differ significantly in terms of optimization coupling strategies, state modeling, and computational complexity, which directly affect their applicability in dynamic mobile-sink scenarios.

The foundation of energy-aware routing lies in heuristic protocols and metaheuristic algorithms. For instance, ref. [1] integrated Ant Colony Optimization (ACO) with Mixed-Integer Linear Programming (MILP) to refine path planning. Subsequent research explored optimal energy scheduling for harvesting-based transmitters [2] and collaborative task offloading strategies [3]. To enhance routing intelligence, Q-learning was introduced to optimize long-term network longevity [4,5]. Furthermore, bio-inspired metaheuristics, such as the Flamingo search algorithm [6], have been leveraged to optimize cluster-head selection. These methods typically rely on heuristic or stochastic search mechanisms, where trajectory planning and routing decisions are implicitly coordinated rather than jointly optimized. While they provide flexibility and scalability, their lack of explicit global–local coupling may lead to suboptimal system-level performance under dynamic conditions.

In the domain of adaptive management, ref. [7] proposed a hybrid K-means and Q-learning approach for node balancing, while [8] utilized MDP models for duty-cycle scheduling. These efforts were complemented by energy-delay reduction models [9,10] and advanced path planning using deep reinforcement learning or adaptive A* search [11,12,13]. Although these approaches introduce adaptive decision-making capabilities, their effectiveness heavily depends on state representation design and learning stability. Moreover, most reinforcement learning-based methods focus on local routing optimization and do not explicitly incorporate global trajectory planning into the decision loop.

Mathematical programming, specifically MILP, remains indispensable for rigorous system-level optimization [14,15,16,17]. Its application extends from medical energy analysis [18] and UAV-assisted data collection [19] to secure routing protocols [20,21]. Recent holistic studies have further integrated WSN coverage with photovoltaic storage and renewable energy systems [22,23,24,25], emphasizing reliability-aware deployment [26,27,28,29,30]. Despite their strong theoretical guarantees, MILP-based approaches are typically designed for static or quasi-static environments and often incur high computational overhead when applied to real-time adaptive scenarios. In addition, they generally lack mechanisms for continuous policy adjustment under dynamic network conditions.

The efficacy of bio-inspired hybrid methodologies has been further substantiated by recent advancements in swarm intelligence and decentralized optimization. For instance, the HACOSMO method [31] demonstrates that the synergistic integration of Ant Colony Optimization (ACO) and Spider Monkey Optimization (SMO) can significantly enhance routing efficiency and adaptability in Vehicular Ad-hoc Networks (VANETs) characterized by multi-constraint requirements. Similarly, hybrid models combining Particle Swarm Optimization (PSO) and Genetic Algorithms (GA) have achieved substantial improvements in energy-efficient computing and resource allocation within intelligent IoT ecosystems, leveraging decentralized self-organization mechanisms to reduce both energy consumption and latency [32]. These hybrid strategies highlight the potential of combining multiple optimization paradigms; however, they still primarily operate within heuristic or metaheuristic frameworks and do not provide a unified mechanism for integrating deterministic optimization with learning-based adaptation.

From a comparative perspective, existing methods can be broadly distinguished by their optimization coupling mechanisms. Heuristic and metaheuristic approaches rely on implicit coordination, reinforcement learning-based methods emphasize local adaptability, while MILP-based approaches provide global optimality under static assumptions. However, most existing hybrid solutions adopt loosely coupled architectures, where trajectory planning and routing adaptation are executed independently or sequentially.

While these bio-inspired frameworks offer robust solutions for static or semi-dynamic environments, certain challenges remain regarding the seamless integration of global path planning and local routing adaptation in highly non-stationary WSNs. Many existing approaches tend to treat sink trajectory selection and real-time data forwarding as decoupled optimization problems, which may lead to suboptimal resource utilization under fluctuating network conditions. Furthermore, while reinforcement learning (RL) offers flexibility, its convergence stability can be sensitive to environmental shifts, whereas traditional Mixed-Integer Linear Programming (MILP) models, despite their mathematical rigor, often face computational bottlenecks in responding to real-time dynamics.

Therefore, a key unresolved issue is the lack of a tightly integrated optimization-learning framework that can simultaneously leverage the global optimality of MILP and the adaptive capability of RL within a unified decision-making process.

Building upon these insights, this study proposes a unified MILP-MDP framework. This approach seeks to complement the global deterministic optimization of MILP with the stochastic adaptive learning of MDPs, aiming to provide a more resilient and energy-balanced solution for dynamic wireless sensor networks.

### 1.3. The Proposed Method

Inspired by the hierarchical decision-making of honeybees, this paper proposes a hybrid MILP-MDP optimization framework.

To address the identified gap of decoupled optimization and adaptation, the proposed method explicitly integrates global trajectory planning with state-aware routing adjustment within a unified decision-making loop.

This dual-layer architecture consists of:

(1) **Global Planning Layer (MILP):** determines the optimal visit sequence of cluster heads.
(2) **Local Adaptive Layer (MDP/Q-learning):** dynamically adjusts routing parameters based on network state.

The algorithm utilizes an ϵ-greedy strategy for balanced exploration–exploitation and incorporates an event-triggered adjustment mechanism to enhance robustness against node failures and energy depletion.

### 1.4. Main Contributions

The primary contributions of this work are as follows:

(1) A tightly coupled MILP-MDP framework is proposed to jointly address global trajectory optimization and local adaptive routing, overcoming the limitations of decoupled approaches.
(2) A multi-objective reward function that explicitly balances network lifetime with end-to-end latency.
(3) An event-triggered adaptive strategy to ensure system resilience.
(4) Extensive simulations demonstrating improved energy balance and network longevity.

### 1.5. Organization

The remainder of this paper is structured as follows: Section 2 defines the system and energy models. Section 3 details the MILP-MDP algorithm. Section 4 presents the simulation analysis. Section 5 discusses the implications of the results, and Section 6 concludes the study.

## 2. System Model

This section describes the system model by studying the structure of the wireless sensor network (WSN), including the network model, energy consumption model, delay model, and dynamic network state representation. These models provide the theoretical foundation for the optimization framework proposed in this paper.

### 2.1. Network Model

We consider a two-dimensional square sensing area of size 100m×100m, in which N=100 static sensor nodes are randomly deployed. Each sensor node is denoted as $S_{i}$, where i=1,2,…,N. All sensor nodes are initialized with a fixed energy of 1.5 J and are not rechargeable during network operation. These parameters (area size, node density, and energy) are selected based on common WSN benchmarks to ensure comparability with existing literature, and their impact is further analyzed in the simulation section.

**Figure 2:**
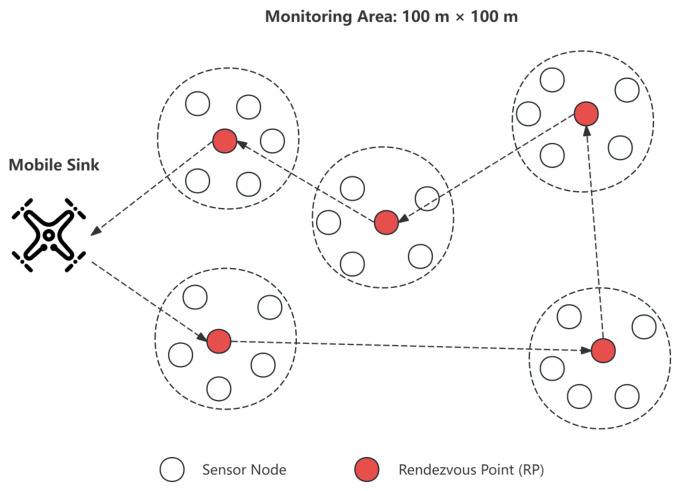
Network model of the mobile-sink-based wireless sensor network.

As illustrated in Figure 2, a mobile sink is deployed in the network and can freely move within the sensing region to collect data from cluster head nodes. The initial position of the sink is set to (50,50), corresponding to the center of the monitoring area, which avoids initial spatial bias, and the sink moves at a constant speed of V=10 m/s.

Each sensor node maintains the following attributes:

(1) Position coordinates $\left(x_{i},y_{i}\right)$;

(2) Residual energy $E_{i}^{t}$ at time *t*;

(3) Node state indicator $cond_{i}\in \left\{0,1\right\}$, where 1 represents an active node and 0 indicates a failed node;

(4) Cluster role, which can be either a cluster head (CH) or a normal member node.

The mobile sink periodically moves within the sensing region and performs global trajectory optimization every ech=10 rounds using the proposed MILP framework. The choice of ech=10 strikes a balance between computational overhead and the system’s responsiveness to energy variations. During each data collection cycle, cluster head nodes aggregate data from their member nodes and forward the collected information to the sink through either single-hop communication or intra-cluster multi-hop transmission.

### 2.2. Energy Consumption Model

In WSNs, node energy consumption mainly arises from three operations: data transmission, data reception, and data aggregation. This study adopts the classical first-order radio energy model to characterize communication energy consumption.

The energy consumption required to transmit a data packet of length *l* bits over a distance *d* is defined as

(1) $$E_{T}\left(l,d\right)=\left\{\begin{array}{cc} lE_{elec}+l\varepsilon_{fs}d^{2}, & d<d_{th}\\ lE_{elec}+l\varepsilon_{mp}d^{4}, & d\geq d_{th} \end{array}\right.$$

The energy consumption required to receive a packet of length *l* bits is given by

(2) $$E_{R}\left(l\right)=lE_{elec}$$

The threshold distance $d_{th}$ between the free-space model and the multipath fading model is calculated as

(3) $$d_{th}=\sqrt{\frac{\varepsilon_{fs}}{\varepsilon_{mp}}}$$

In addition, cluster head nodes perform data aggregation before forwarding packets to the sink. The corresponding energy consumption is expressed as

(4) $$E_{DA}\left(l\right)=lE_{DA}$$

In this work, the following parameter values are adopted: the packet size is l=4000 bits, the electronic energy consumption for transmission and reception is $E_{elec}=50$ nJ/bit, the free-space channel coefficient is $\varepsilon_{fs}=10$ pJ/bit/m^2^, and the multipath fading coefficient is $\varepsilon_{mp}=0.0013$ pJ/bit/m^4^. The data aggregation energy is set to $E_{DA}=5$ nJ/bit. These values are consistent with the classic radio model parameters and can be adjusted to represent different hardware specifications without loss of generality.

According to (1), communication energy consumption increases rapidly with transmission distance, especially under multipath propagation conditions. This model effectively captures the nonlinear relationship between communication distance and node energy consumption in WSNs.

### 2.3. Delay Model

The total system delay consists of two components: the sink movement delay and the data transmission delay. The total delay is defined as

(5) $$T_{total}=\frac{d_{sink}}{V}+\frac{D_{sum}\;L}{R}$$

where $d_{sink}$ denotes the traveling distance of the mobile sink in the current round, V=10 m/s represents the sink movement speed, L=4000 bits denotes the packet size, $D_{sum}$ represents the total number of transmitted packets, and R=250 kbit/s denotes the data transmission rate. This analytical decomposition allows the optimization framework to evaluate the impact of trajectory length on real-time data delivery.

This delay model reflects the coupling relationship between sink mobility and communication transmission. Shorter trajectories can reduce movement delay but may increase communication distances, which in turn leads to higher energy consumption. Therefore, trajectory optimization must consider the trade-off between energy efficiency and communication delay.

### 2.4. Spatiotemporal Data Correlation

In practical WSN applications, sensor data generation usually exhibits both temporal randomness and spatial correlation. To capture these characteristics, the data generation process is modeled using a spatiotemporal stochastic model.

The temporal variation in data generation is modeled using a Poisson distribution:

(6) $$D_{i}∼\mathrm{Poisson}\left(\lambda_{time}\right)$$

where $\lambda_{time}=5$ represents the average data generation rate.

To reflect spatial heterogeneity in the environment (e.g., localized temperature or pressure spikes), the data volume is further adjusted using a spatial weighting function that is independent of the sink’s location:

(7) $$D_{i}^{*}=D_{i}\cdot e^{-\alpha d_{ie}}$$

where $d_{ie}$ denotes the distance between node *i* and a randomly assigned environmental “event center,” and α=0.1 is the spatial correlation coefficient. This modeling approach captures the combined effects of temporal dynamics and spatial distribution on sensor data generation in WSN environments.

### 2.5. Event-Triggered Network State Update

Wireless sensor networks operate in dynamic environments where node energy levels, network topology, and traffic loads may change over time. To reduce computational overhead, an event-triggered update mechanism is adopted instead of continuously recomputing routing strategies.

To provide a comprehensive representation of the network’s dynamic context, the network state at time *t* is defined as a four-dimensional vector:

(8) $$S_{t}=\left(E_{avg},A_{ratio},L_{data},D_{sink}\right)$$

where $E_{avg}$ denotes the average residual energy of all nodes, $A_{ratio}$ represents the proportion of active nodes, $L_{data}$ indicates the current data load, and $D_{sink}$ represents the average distance from nodes to the current sink position. By discretizing each dimension into 3 levels, the state space consists of $3^{4}=81$ states, ensuring efficient Q-learning convergence.

An optimization update is triggered only when the change in network state exceeds a predefined threshold:

(9) $${∥{\hat{S}}_{t}-{\hat{S}}_{t-1}∥}_{2}>\delta$$

where $\hat{S}$ denotes the normalized state vector, δ denotes the event-trigger threshold, and ${∥\cdot ∥}_{2}$ represents the Euclidean norm.

This mechanism ensures that the mobile sink adjusts its routing strategy only when significant network changes occur, thereby improving computational efficiency while maintaining adaptive performance. The event-triggered update mechanism will be integrated into the adaptive optimization framework presented in the next section.

## 3. MILP-MDP Based Adaptive Routing Algorithm

This section presents an adaptive routing framework for mobile-sink-assisted wireless sensor networks (WSNs). The proposed framework integrates mixed-integer linear programming (MILP) with a Markov decision process (MDP)-based reinforcement learning mechanism, aiming to optimize routing decisions under dynamically evolving network conditions.

In contrast to loosely coupled hybrid methods, an explicit interaction loop is established between the MILP optimization layer and the MDP learning layer. The deterministic output of MILP serves as the strategic baseline, which is then refined by the Q-learning agent based on real-time network feedback.

The proposed method employs a hierarchical architecture. At the upper layer, the MILP model determines the globally optimal visiting sequence of cluster heads, providing a baseline routing solution for the mobile sink. At the lower layer, a Q-learning-based agent continuously interacts with the network environment and adaptively adjusts routing parameters according to the observed system state. This combination enables the routing strategy to evolve over time, thereby improving long-term performance in terms of energy efficiency, delay, and network lifetime.

### 3.1. Overall Framework

The routing process operates in discrete rounds. During each round, the network state is updated, and routing decisions are periodically adjusted according to a predefined decision interval.

**Figure 3:**
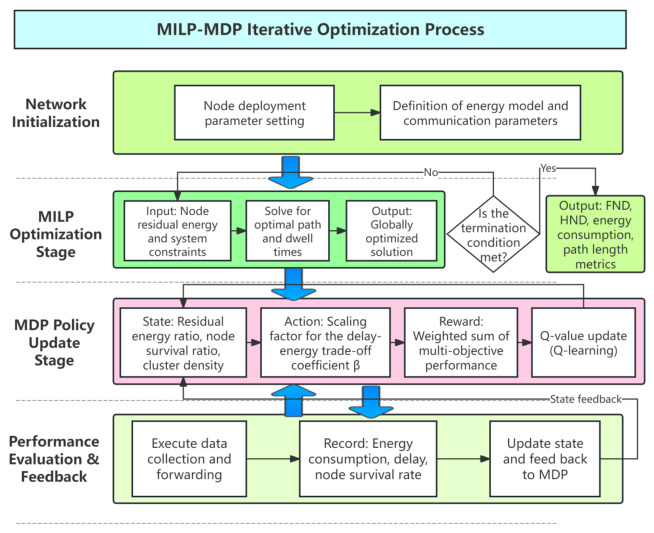
Overall workflow of the proposed MILP-MDP adaptive routing framework.

Figure 3 illustrates the overall workflow of the proposed framework. First, clustering is performed to identify cluster heads that act as rendezvous points for data collection.

In this study, a Distance–Energy Weighted K-means mechanism is adopted for clustering. Specifically, the clustering process jointly considers the Euclidean distance between nodes and cluster centers and the residual energy of nodes. The weighted distance metric is defined as

(10) $$D_{i}=\lambda d_{i}+\left(1-\lambda \right)\frac{1}{E_{i}}$$

where $d_{i}$ denotes the Euclidean distance to the cluster centroid, $E_{i}$ represents the residual energy of node *i*, and λ∈[0,1] is a balancing coefficient. This formulation prioritizes nodes with higher residual energy and shorter distances for cluster-head selection, thereby reducing the likelihood of premature energy depletion.

To ensure fairness in comparative experiments, the same clustering results are shared across all evaluated algorithms, so that performance differences arise from routing strategies rather than clustering variations.

Based on the current network configuration, the MILP model computes the optimal visiting sequence and dwell time of the mobile sink. Subsequently, the MDP agent observes the network state and selects an action to scale the routing solution generated by the MILP model. This adaptive adjustment enables the system to respond to dynamic variations such as energy depletion and topology changes.

In contrast to conventional MILP-based approaches that assume static network conditions, the proposed framework explicitly accounts for the time-varying nature of WSNs. As the network evolves, node energy decreases, some nodes may fail, and traffic patterns may fluctuate. Therefore, a static routing plan becomes suboptimal over time. The integration of reinforcement learning provides the capability to dynamically refine routing decisions, ensuring sustained performance throughout the network lifetime.

The action space of the MDP agent consists of four scaling factors:

(11) A={0.8,0.9,1.0,1.1}

Each action modifies the execution intensity of the MILP-derived routing plan, thereby enabling adaptive control of sink movement and dwell time.

### 3.2. MILP-Based Path Optimization

Let RP={1,2,…,k} denote the set of cluster heads. The MILP model determines the visiting sequence and corresponding dwell times of the mobile sink.

The objective function is defined as

(12) $$\min\sum_{(i,j)\in E}\left[\left(1-\gamma \right)\frac{d_{i,j}}{V}+\gamma d_{i,j}\right]x_{i,j}+\sum_{i\in RP}\beta \frac{lD_{i}}{RD_{\max}}t_{i}$$

where γ∈[0,1] controls the trade-off between movement time and travel distance. The parameter $x_{i,j}$ is a binary decision variable indicating whether the sink moves from cluster head *i* to *j*, while $t_{i}$ denotes the dwell time at cluster *i*.

The Euclidean distance is given by

(13) $$d_{i,EC}=\sqrt{{\left(X_{EC}-X_{i}\right)}^{2}+{\left(Y_{EC}-Y_{i}\right)}^{2}}$$

To ensure the mathematical completeness and reproducibility of the routing model, the following constraints are explicitly defined:

(14) $$\sum_{j\in RP,j\neq i}x_{i,j}=1,\;\forall i\in RP$$

(15) $$\sum_{i\in RP,i\neq j}x_{i,j}=1,\;\forall j\in RP$$

(16) $$u_{i}-u_{j}+kx_{i,j}\leq k-1,\;\forall i,j\in \left\{2,\ldots ,k\right\},i\neq j$$

(17) $$t_{i}\geq \frac{L_{i}}{R},\;\forall i\in RP$$

where the first two equations represent flow conservation, the third is the Miller–Tucker–Zemlin (MTZ) constraint for subtour elimination, and the last ensures sufficient dwell time for data collection. The solution yields the total path length $P_{length}$ and total delay $T_{delay}$.

To incorporate adaptability, the routing delay is adjusted by a scaling factor determined by the MDP:

(18) $$T_{effective}=scale\cdot T_{delay}$$

### 3.3. MDP State Representation

The system state is defined as a four-dimensional vector following the standard formulation of Markov Decision Process (MDP) models:

(19) $$s=(s_{E},s_{A},s_{D},s_{C})$$

which captures key characteristics of the network.

(1) The energy component is defined using the global energy ratio:

(20) $$E_{ratio}=\frac{\sum_{i=1}^{N}E_{i}}{NE_{0}}$$

This formulation reflects the overall degradation of the network and accounts for dead nodes by assigning zero energy.

(2) The survivability component is represented by the alive-node ratio:

(21) $$A_{ratio}=\frac{N_{alive}}{N}$$

(3) The data load component corresponds to the average data generation rate of alive nodes at the current time period, normalized to ensure consistency across different traffic conditions.

(4) The cluster density is defined as

(22) $$C_{ratio}=\frac{k}{N}$$

To reduce computational complexity, each state variable is discretized into three levels:

(23) $$s=\left\{\begin{array}{cc} 1, & x<0.33\\ 2, & 0.33\leq x<0.66\\ 3, & x\geq 0.66 \end{array}\right.$$

The thresholds 0.33 and 0.66 are chosen to evenly partition the normalized state space, providing a coarse yet effective abstraction of network conditions. This discretization enables the system to distinguish between low, medium, and high regimes for each state variable, which is sufficient to capture major transitions in network dynamics.

In addition, preliminary sensitivity experiments with alternative discretization schemes (e.g., binary and four-level partitions) were conducted, showing no significant improvement in performance but a noticeable increase in convergence time. Therefore, the three-level discretization is adopted as a trade-off between representational capacity and learning efficiency, resulting in a compact yet expressive state space of size $3^{4}=81$.

### 3.4. Reward Function Design

The reward function plays a central role in guiding the reinforcement learning agent’s decision-making process. In the context of WSN routing, multiple performance objectives must be considered simultaneously, including energy efficiency, network lifetime, communication delay, and routing cost. These objectives are often conflicting; for example, reducing delay may require faster sink movement, which can increase energy consumption.

To address this multi-objective optimization problem, a composite reward function is constructed as

(24) $$R=w_{E}E_{norm}+w_{A}A_{ratio}-w_{D}D_{norm}-w_{P}P_{norm}$$

where the weighting coefficients satisfy $w_{E}+w_{A}+w_{D}+w_{P}=1$.

The individual components are defined as follows.

(1) The energy term is defined as

(25) $$E_{norm}=\frac{E_{total}}{E_{initial}}$$

where $E_{total}$ represents the total residual energy of all nodes. Compared with average energy metrics, this formulation provides a more accurate global representation of network status and naturally incorporates the effect of node failures.

(2) The survivability term is given by

(26) $$A_{ratio}=\frac{N_{alive}}{N}$$

This term directly reflects network connectivity and sensing coverage, encouraging routing strategies that delay node death and prolong network lifetime.

(3) The delay term is defined as

(27) $$D_{norm}=\frac{Delay}{Delay_{max}}$$

which penalizes excessive communication latency. The normalization ensures numerical stability during the learning process.

(4) The routing cost term is expressed as

(28) $$P_{norm}=\frac{Path_{length}}{Path_{max}}$$

This term discourages unnecessarily long sink trajectories, which would otherwise increase both delay and energy consumption.

The structure of the reward function reflects a balance between maximization objectives (energy preservation and survivability) and minimization objectives (delay and routing cost). By integrating these factors into a single scalar value, the reinforcement learning agent can evaluate the long-term effectiveness of routing decisions.

Furthermore, the weighting coefficients are not fixed a priori but determined through a sensitivity analysis procedure. Different weight combinations are evaluated, and the set that maximizes the performance score is selected. This mechanism enables the model to adaptively emphasize more critical objectives under varying network conditions, thereby enhancing robustness and generalization capability.

Overall, the proposed reward function provides a unified and scalable formulation that captures the essential performance trade-offs in WSN routing and facilitates efficient policy learning.

### 3.5. Q-Learning Policy Update

In order to enable adaptive routing decisions under dynamic network conditions, a Q-learning algorithm is employed to learn the optimal policy through continuous interaction with the environment.

The Q-value update rule is defined as

(29) $$Q\left(s,a\right)\leftarrow \left(1-\alpha_{q}\right)Q\left(s,a\right)+\alpha_{q}\left[R+\gamma_{q}\max_{a^{\prime }}Q\left(s^{\prime },a^{\prime }\right)\right]$$

where $\alpha_{q}$ denotes the learning rate, $\gamma_{q}$ represents the discount factor, *R* is the immediate reward, and $s^{\prime }$ is the next state. This update mechanism allows the agent to iteratively refine its estimation of long-term returns by combining immediate feedback with future rewards.

To balance exploration and exploitation, an ε-greedy strategy is adopted. Specifically, at each decision step, the agent selects a random action with probability ε, while with probability 1−ε, it selects the action that maximizes the current Q-value. This mechanism ensures that the agent explores the action space sufficiently while gradually exploiting learned knowledge.

In this study, the initial exploration rate is set to ε=0.3. This value is selected based on commonly adopted empirical ranges in reinforcement learning (typically between 0.2 and 0.4), which provide a suitable trade-off between exploration and convergence stability. A relatively higher initial exploration rate allows the agent to sufficiently sample different routing strategies during the early learning stage, thereby avoiding premature convergence to suboptimal policies.

To further improve learning efficiency, an exponential decay strategy is applied to the exploration rate:

(30) ε=max(0.05,ε×0.995)

This decay mechanism ensures that the agent performs extensive exploration in the early stages of training, while gradually shifting toward exploitation as the learning process progresses. The lower bound of 0.05 is introduced to maintain a minimal level of exploration, preventing the agent from being trapped in local optima.

Through the combination of Q-value updates and adaptive exploration, the proposed learning mechanism enables the agent to converge toward an effective routing policy that balances energy efficiency, delay, and network lifetime.

### 3.6. Algorithm Workflow

The overall procedure of the proposed MILP-MDP routing algorithm is summarized in Algorithm 1. At each round, the network state is first updated according to the residual energy and node conditions. When a decision epoch is reached, clustering is performed and the MILP model is invoked to compute the routing path. The MDP agent then observes the current state and selects an action using the ε-greedy strategy. The routing plan is subsequently adjusted based on the selected action. After data transmission, the reward is calculated, and the Q-table is updated accordingly. Through repeated iterations, the agent gradually learns an optimal routing policy.
**Algorithm 1** MILP-MDP adaptive routing algorithm**Input:** Network parameters, $E_{0}$, *V*, α, β, γ, packetLength**Output:** Network lifetime, delay, FND, HNDInitialize Q-table Q(s,a) for 81 statesInitialize exploration rate ε=0.3Define action set A={0.8,0.9,1.0,1.1}**for** each round *r* **do**   Update node energy and alive-node status   **if** r=1 or rmodech=0 or event-triggered **then**     Perform Distance–Energy Weighted K-means clustering     Observe current state s∈{1,…,81}     Select action *A* using ε-greedy policy     Solve MILP to obtain routing path subject to flow and subtour constraints     Apply scaling factor to adjust routing   **end if**   Execute data transmission   Compute reward *R* according to Equation (24)   Observe next state $s^{\prime }$   Update Q-table using Q-learning rule   Update exploration rate ε**end for**Record performance metrics (FND, HND, delay, energy)

## 4. Experiments

In this section, extensive simulation experiments are conducted to evaluate the effectiveness of the proposed MILP-MDP adaptive routing framework for mobile-sink-based wireless sensor networks (WSNs). The primary objective is to investigate whether integrating reinforcement learning with deterministic optimization can further improve routing performance and energy efficiency.

For comprehensive comparison, the proposed method is evaluated against several representative algorithms, including IS-kmeans clustering, SOGA-AESA evolutionary optimization, the classical MILP routing model, and a MILP variant without adaptive learning (MILP-noMDP). The proposed MILP-MDP algorithm incorporates a Q-learning-based Markov Decision Process (MDP) that dynamically adjusts routing weights according to network conditions.

To ensure fairness and reproducibility, all baseline algorithms are re-implemented within the same simulation environment using a unified network configuration, including identical node deployment, energy model, and traffic assumptions. In addition, all methods operate on the same clustering results generated by the Distance–Energy Weighted K-means mechanism, and their key parameters are tuned according to commonly adopted settings in the literature. This ensures that performance differences arise from routing strategies rather than inconsistencies in experimental conditions.

Three widely used performance metrics are adopted for evaluation: network lifetime, communication delay, and energy efficiency.

### 4.1. Simulation Parameter Settings

The simulation environment is implemented in MATLAB R2018b, where all algorithms are executed on the same platform to ensure a fair comparison. The MILP optimization problem is solved using MATLAB’s built-in intlinprog solver. The simulation parameters are summarized in Table 1.

### 4.2. Computational Complexity and Real-Time Feasibility

To verify the practical feasibility of the proposed framework, the average computational time for 1500 simulation rounds was measured. As shown in Table 2, the proposed MILP-MDP framework completes the task in approximately 3.4 s. Although the MILP problem is inherently NP-hard, the search space for the MILP solver is restricted to a extremely small range by limiting the scale of the sink capacity ($k_{cap}\leq 20$). The evidence from the simulation logs (MILP-MDP done in 3.5 s) confirms that the algorithm possesses the potential for on-line implementation without causing computational bottlenecks at the sink node.

### 4.3. Network Lifetime Analysis

Network lifetime is a fundamental metric for evaluating WSN performance. In this study, two commonly used indicators are adopted: the First Node Death (FND) and the Half Node Death (HND).

**Figure 4:**
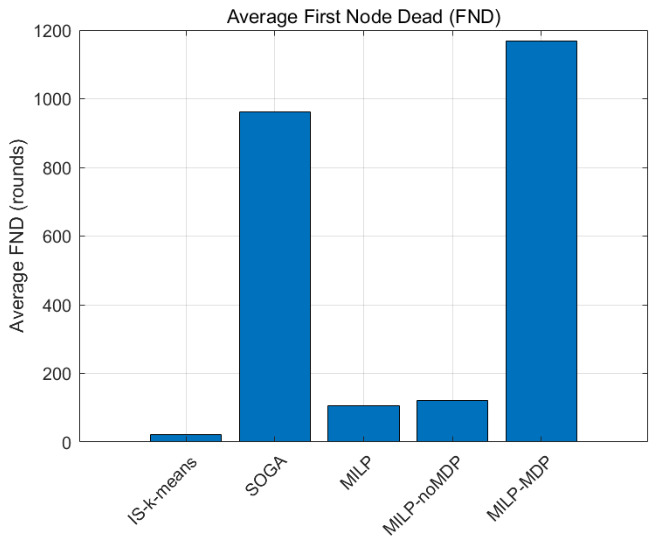
Comparison of the average First Node Death (FND) among different algorithms.

Figure 4 illustrates the average FND results of different algorithms, while the detailed numerical comparison is presented in Table 3.

The experimental results indicate that the proposed MILP-MDP framework significantly outperforms all comparison algorithms in terms of network lifetime. Specifically, the proposed method achieves an average FND of 972.3 rounds, approximately 10.1 times that of the classical MILP routing model (statistical significance $p<10^{-6}$).

It can also be observed that MILP-noMDP provides only limited improvement compared with the original MILP approach. This suggests that relying solely on static optimization strategies is insufficient to maintain balanced energy consumption throughout the network lifecycle. By incorporating the MDP-based reinforcement learning mechanism, the system can dynamically adjust routing parameters based on real-time network states, thereby effectively mitigating local energy hotspots.

Although SOGA-AESA benefits from global evolutionary search capabilities and achieves relatively long lifetimes, it lacks the ability to adapt routing decisions dynamically according to current network conditions. In contrast, the proposed MILP-MDP framework continuously optimizes routing strategies through reinforcement learning, resulting in more balanced energy consumption and delayed node failures.

**Figure 5:**
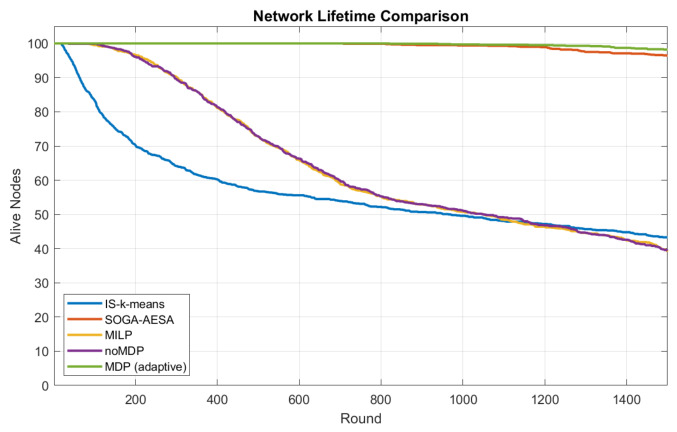
Number of alive nodes versus simulation rounds for different algorithms.

Figure 5 further shows the variation in the number of alive nodes over simulation rounds. The proposed algorithm maintains the largest number of active nodes for the longest duration, confirming its superior lifetime performance.

### 4.4. Communication Delay Analysis

Communication delay reflects the efficiency of data collection in mobile-sink-based WSNs. Table 4 presents the comparison of average end-to-end communication delays among the evaluated algorithms.

As shown in Table 4, the proposed MILP-MDP algorithm reduces the average delay from 3871.9 ms (MILP) to 3586.6 ms, demonstrating a noticeable improvement over traditional deterministic optimization methods. A *t*-test ($p<10^{-6}$) confirms the statistical significance of this improvement.

The reduction in delay mainly results from the adaptive adjustment of routing weights and dwell times, enabling the mobile sink to select more efficient data collection paths according to the current network state. Although SOGA-AESA achieves the lowest delay, this advantage is obtained at the cost of energy imbalance and longer routing paths, which ultimately affects long-term network sustainability. Therefore, the proposed MILP-MDP method achieves a better balance between communication efficiency and energy sustainability.

### 4.5. Energy Efficiency Analysis

**Figure 6:**
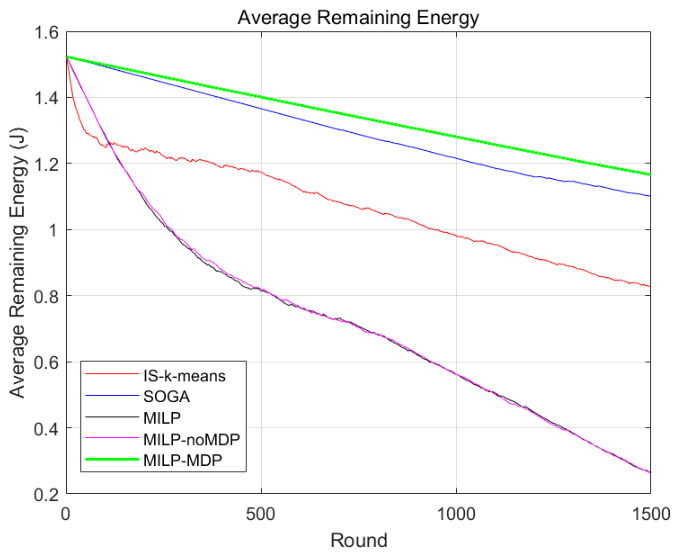
Average residual network energy over time for different algorithms.

Energy efficiency directly affects the operational lifetime of WSNs. Figure 6 shows the variation of the average residual network energy over simulation rounds for different algorithms.

The results in Table 5 show that the proposed algorithm maintains the highest residual energy among all evaluated methods. The slower decline in energy consumption indicates that the adaptive routing strategy effectively distributes communication loads across the network, thereby mitigating the formation of energy hotspots. This confirms that integrating reinforcement learning with deterministic optimization significantly improves the overall energy utilization efficiency of the network.

### 4.6. Scalability Analysis

**Figure 7:**
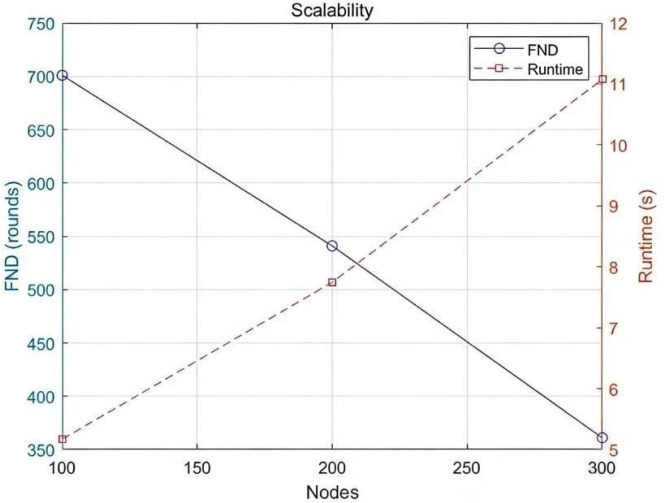
Variation of the FND metric with increasing network size.

To further evaluate the scalability of the proposed framework, additional simulations were conducted by increasing the number of sensor nodes from 100 to 300 while keeping other parameters unchanged. Figure 7 illustrates the variation of the FND metric with increasing network size.

The results indicate that as the number of nodes increases, the network lifetime gradually decreases from approximately 700 rounds to 545 rounds and further to 355 rounds. This trend is expected because higher node density intensifies communication contention and relay load, thereby accelerating energy consumption.

Despite this reduction, the proposed MILP-MDP framework still maintains relatively long network lifetimes, demonstrating its ability to adapt to increasing network density. In addition, the computational complexity of the MILP optimization increases with network size, leading to moderate growth in execution time. Nevertheless, the increase remains manageable, indicating that the proposed method is practical for medium-scale WSN deployments.

### 4.7. Reinforcement Learning Behavior Analysis

**Figure 8:**
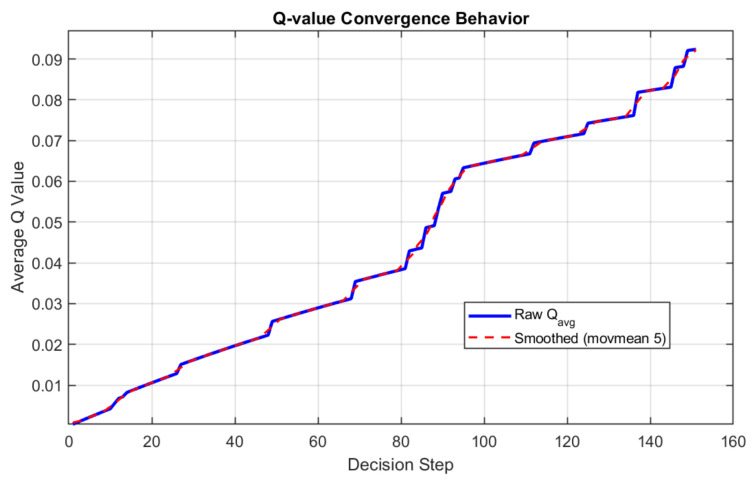
Evolution of the average Q-value during routing decision making.

To examine the learning dynamics of the proposed MILP-MDP framework, the evolution of the Q-learning process is analyzed. Figure 8 shows the variation of the average Q-value over routing decision steps.

The average Q-value is computed over all state-action pairs in the Q-table at each decision step, providing a global measure of the expected cumulative reward.

As observed in Figure 8, the average Q-value increases steadily as the number of decision steps grows. This trend indicates that the reinforcement learning agent progressively improves its policy through interaction with the network environment. Actions that yield higher rewards, such as those improving energy efficiency and reducing delay, are reinforced during the learning process, leading to higher Q-value estimates over time.

The smoothed curve further confirms that this trend is consistent rather than caused by short-term fluctuations, suggesting stable learning behavior. Although a clear plateau is not observed within the considered decision horizon, the continuous increase implies that the agent is still refining its routing strategy.

Such behavior is reasonable in dynamic WSN scenarios, where network conditions evolve due to energy depletion and node failures. As a result, the learning process reflects ongoing adaptation rather than strict convergence to a fixed policy.

Overall, the results demonstrate that the proposed reinforcement learning mechanism effectively improves routing decisions while maintaining stable and adaptive behavior in a time-varying environment.

### 4.8. Sensitivity Analysis of Sink Capacity ($k_{cap}$)

To investigate the impact of the sink’s collection capacity on network performance, a sensitivity analysis was conducted with $k_{cap}$ values of 5, 10, 15, and 20. The collection capacity defines the maximum number of data packets the mobile sink can receive at a specific anchor point before moving. As summarized in Table 6, the results reveal a significant non-linear relationship between $k_{cap}$ and system metrics.

The experimental data identifies $k_{cap}=10$ as the optimal “sweet spot” for the considered WSN environment. When the capacity is restricted ($k_{cap}=5$), the system suffers from severe data backlogs and frequent sink migrations, leading to an extremely high delay of 17,492.5 ms and a premature FND at round 221 due to energy exhaustion in relay nodes.

Interestingly, increasing the capacity beyond 10 (e.g., $k_{cap}=15$ or 20) does not yield monotonic improvements in network lifetime. While larger capacities can further reduce communication latency by allowing longer dwell times at optimal locations, they tend to concentrate the relay load on specific neighborhood nodes for extended periods, resulting in uneven energy depletion. Specifically, the FND drops to 491 and 561 rounds for $k_{cap}=15$ and 20, respectively. Therefore, $k_{cap}=10$ is selected as the baseline parameter as it provides the most robust balance between maximizing network longevity and maintaining acceptable communication delays.

### 4.9. Overall Performance Discussion

Overall, the simulation results demonstrate that the proposed MILP-MDP framework significantly outperforms traditional routing algorithms in terms of network lifetime and energy efficiency. Supported by a high-efficiency execution (3.5 s per 1500 rounds) and statistically significant improvements ($p<10^{-6}$), the proposed method dynamically adjusts routing strategies according to the network state.

The comparison with MILP-noMDP further highlights the importance of the reinforcement learning component. Combined with the scalability across 100 to 300 nodes and the sensitivity analysis of $k_{cap}$, the introduction of the MDP learning mechanism enables continuous adaptation based on node energy levels, traffic loads, and topology variations.

Although evolutionary approaches such as SOGA-AESA may achieve lower communication delays, they often suffer from energy imbalance. In contrast, the proposed framework provides a more robust and sustainable routing strategy while maintaining acceptable communication delays. Therefore, dynamic adaptation emerges as the key mechanism driving performance improvement rather than the result of initial optimization alone.

## 5. Discussion

This section analyzes the behavior and implications of the proposed MILP-MDP adaptive optimization framework based on the simulation results and the algorithmic mechanisms implemented in the model. The discussion focuses on the observed performance characteristics, the interaction between the optimization and learning components, and potential research extensions for large-scale wireless sensor networks.

### 5.1. Key Findings

The simulation results provide strong evidence that the proposed hybrid MILP-MDP framework effectively balances multiple performance objectives in wireless sensor networks, including energy efficiency, node survival ratio, data collection delay, and mobile sink trajectory cost. Unlike traditional routing approaches that rely solely on static optimization or purely learning-based strategies, the proposed framework integrates deterministic optimization with adaptive decision learning.

Specifically, the MILP component performs global path planning for the mobile sink by optimizing the visiting sequence of rendezvous points under delay-related constraints. The optimization process outputs the path length and data collection delay associated with the selected trajectory. These outputs are further utilized as quantitative feedback signals for the MDP layer, enabling adaptive refinement of the routing strategy based on real-time network conditions.

The MDP module models the network condition using a compact discrete state representation composed of four factors: the global residual energy ratio, the proportion of alive nodes, the average sensing data load, and the cluster ratio. Each dimension is discretized into three levels, resulting in a state space of $3^{4}=81$ possible states. This compact representation provides a balance between descriptive capability and computational efficiency, allowing the system to capture essential network dynamics without introducing excessive learning complexity.

At each decision interval, the reinforcement learning agent selects an action from a predefined action set to scale delay-related coefficients in the MILP formulation. The action set {0.8,0.9,1.0,1.1} modifies the effective optimization parameters controlling the trade-off between delay and trajectory cost in the MILP model. Through this mechanism, the system dynamically adapts the mobile sink trajectory planning strategy according to the current network state.

The reward function integrates multiple performance indicators, including normalized total network energy, node survival ratio, normalized delay, and normalized path length. These indicators are combined through weighted aggregation to generate a scalar reward value used for guiding the Q-learning update process. This multi-objective design enables the learning agent to balance conflicting objectives, such as minimizing delay while preserving long-term network energy. The reward weights are determined through a sensitivity-based selection procedure, in which candidate weight configurations are evaluated and the configuration producing the highest estimated performance score is selected as the final reward setting.

Simulation observations indicate that this adaptive mechanism allows the system to gradually learn suitable parameter-scaling strategies for different network conditions. When energy availability decreases or the proportion of alive nodes declines, the learning module tends to select actions that reduce trajectory cost and energy consumption. Conversely, when energy resources are abundant, the framework emphasizes delay reduction through more aggressive path optimization.

Another important observation concerns the role of the exploration mechanism in stabilizing the learning process. The proposed algorithm employs an exponentially decaying ε-greedy strategy that enables the learning agent to explore diverse parameter scaling options during early stages and gradually shift toward exploiting high-reward strategies as training progresses. This mechanism helps maintain learning stability and prevents premature convergence to suboptimal decisions.

Furthermore, the decision process is executed only at specific decision intervals rather than at every round. Between two decision points, previously obtained delay and path results are reused. This periodic decision strategy significantly reduces unnecessary computational overhead by avoiding frequent optimization calls, thereby improving the practical feasibility of the framework during long-term network operation.

It is also important to note that the proposed framework does not universally dominate all performance metrics. For example, the SOGA-AESA algorithm achieves lower communication delay in certain scenarios due to its aggressive trajectory optimization strategy. However, this improvement is accompanied by uneven energy consumption and reduced long-term network stability. This observation highlights a fundamental trade-off between short-term efficiency and long-term sustainability.

Furthermore, under extremely dense network conditions, the MILP component may experience increased computational overhead due to the expansion of the optimization search space, which could limit real-time responsiveness if not properly constrained. These observations indicate that while the proposed framework achieves a balanced performance in typical scenarios, its advantages are most pronounced in environments requiring adaptive and energy-aware decision-making.

Overall, the simulation results confirm that integrating reinforcement learning with MILP optimization enables adaptive trajectory planning for the mobile sink while maintaining balanced energy consumption and stable network operation.

### 5.2. Future Research Directions

Although the proposed framework demonstrates promising performance, several research directions remain open for further investigation.

First, scalability in large-scale wireless sensor networks remains an important challenge. The current framework relies on centralized MILP optimization for trajectory planning. Although the reinforcement learning module reduces the need for frequent re-optimization, the computational burden of solving MILP problems may increase significantly as the number of rendezvous points grows. Future work could explore decomposition-based MILP formulations or hierarchical optimization strategies to further improve scalability.

Second, improvements in state representation and learning efficiency deserve further attention. The current reinforcement learning module adopts a discrete state space constructed from four network indicators. While this design ensures fast convergence and stability, more expressive representations may capture finer-grained variations in network dynamics. Future studies may investigate continuous-state reinforcement learning methods or function approximation techniques to enhance learning efficiency while maintaining algorithmic stability.

Third, robustness under uncertain network conditions is another important direction. The current model assumes deterministic communication and sensing conditions during simulation. In practical wireless sensor networks, stochastic factors such as packet loss, fluctuating communication quality, and environmental interference may affect system performance. Incorporating stochastic elements into the optimization and learning processes could provide a more realistic evaluation of the framework under uncertain operating conditions.

Addressing these issues would further enhance the applicability of the proposed MILP-MDP framework in real-world large-scale sensor network environments.

### 5.3. Practical Implications

From a practical deployment perspective, the proposed adaptive optimization framework provides several advantages for real-world wireless sensor network applications.

First, the integration of optimization and learning enables flexible adaptation to changing network conditions without requiring frequent manual parameter tuning. The adaptive interaction between MILP-based planning and MDP-based decision-making allows the system to dynamically adjust routing behavior according to evolving network states. Network operators can deploy the framework with predefined parameter ranges while allowing the reinforcement learning module to automatically adjust optimization behavior based on observed network performance.

Second, the use of global energy ratio and node survival ratio in the decision state representation allows the system to effectively monitor the overall health of the network. This enables proactive adjustment of the mobile sink trajectory in response to early signs of node failure or energy depletion, thereby preventing abrupt network collapse.

Third, the periodic decision mechanism significantly reduces computational overhead while preserving adaptive optimization capability. This design improves the feasibility of deploying the framework on edge servers or centralized controllers with limited computational resources. This makes it suitable for long-term monitoring applications.

These characteristics make the proposed framework applicable to various wireless sensor network scenarios, including environmental monitoring, smart agriculture, and industrial sensing systems, where long network lifetime and reliable data collection are essential requirements.

## 6. Conclusions

To address the challenges of trajectory planning and energy management for mobile sinks in wireless sensor networks (WSNs), this study proposes an adaptive optimization framework that integrates Mixed-Integer Linear Programming (MILP) with reinforcement learning (RL). The proposed methodology maps the dual-layer decision-making mechanism observed in honeybee foraging behavior onto a synergistic architecture comprising MILP-based global trajectory optimization and Markov Decision Process (MDP)-driven local adaptive adjustments. This approach offers a potential solution for the joint optimization of latency control, energy efficiency, and network longevity.

Within the proposed framework, the MILP module generates a global initial trajectory for the mobile sink, while the MDP-based RL module adaptively tunes routing policies by dynamically scaling latency-related parameters within the MILP formulation. The learning process is guided by a multi-objective reward function that incorporates critical metrics, including global residual energy, the ratio of surviving nodes, data collection latency, and path length. Furthermore, the design of a discrete state representation captures essential network dynamics while maintaining manageable computational complexity for the learning process.

Simulation results demonstrate that the proposed framework effectively adapts the trajectory planning strategy of the mobile sink in response to network dynamics. Through the synergy of global optimization and local self-learning, the system achieves a balanced trade-off between energy utilization efficiency, end-to-end latency, and node survival rates. The introduction of a periodic decision-update mechanism further enhances computational efficiency by reducing redundant optimization calls.

It should be noted that the proposed framework possesses certain limitations. Future research will focus on scalability issues in large-scale sensor networks, refinement of state representations for reinforcement learning, and the modeling of stochastic network conditions. Overall, this study represents a preliminary attempt to integrate optimization and learning for mobile sink scheduling in dynamic WSNs, providing a reference for adaptive data collection strategies in resource-constrained environments.

## Figures and Tables

**Table 1:** Parameter settings.

Parameter	Value
Simulation area	100m×100m
Number of nodes (*N*)	100
Initial energy ($E_{0}$)	1.5 J
Energy consumption per bit ($E_{elec}$)	50 nJ/bit
Data aggregation energy ($E_{DA}$)	5 nJ/bit
Free-space coefficient ($\epsilon_{fs}$)	10 pJ/bit/m^2^
Multipath coefficient ($\epsilon_{mp}$)	0.0013 pJ/bit/m^4^
Data packet size	4000 bit
Mobile sink speed (*V*)	10 m/s
Communication range ($R_{C}$)	50 m
Path length penalty weight (γ)	0.3
Spatio-temporal correlation parameter (α)	0.1
Residence time weight (β)	0.5
Time correlation parameter ($\lambda_{time}$)	5
Sink capacity ($k_{cap}$)	10

**Table 2:** Average computational runtime comparison.

Algorithm	Runtime (s)
IS-kmeans	2.2
SOGA-AESA	21.8
MILP	7.7
MILP-noMDP	7.0
MILP-MDP (Proposed)	3.4

**Table 3:** Network lifetime comparison.

Algorithm	FND (Rounds)	HND (Rounds)
IS-kmeans	24.0±0.2	964.7±10.0
SOGA-AESA	709.7±24.6	1500.0±0.0
MILP	96.1±2.5	992.3±10.3
MILP-noMDP	106.1±3.3	996.3±10.1
MILP-MDP (Proposed)	972.3±23.4	1500.0±0.0

**Table 4:** Average communication delay comparison.

Algorithm	Average Delay (ms)
IS-kmeans	1645.0±38.4
SOGA-AESA	609.8±48.0
MILP	3871.9±2824.7
MILP-noMDP	3835.2±2733.0
MILP-MDP (Proposed)	3586.6±1092.0

**Table 5:** Average residual network energy comparison.

Algorithm	Average Residual Energy (J)
IS-kmeans	1.070±0.012
SOGA-AESA	1.296±0.131
MILP	0.726±0.306
MILP-noMDP	0.728±0.309
MILP-MDP (Proposed)	1.342±0.111

**Table 6:** Performance sensitivity analysis for various $k_{cap}$ configurations.

$k_{\mathrm{cap}}$	FND (Rounds)	Maximum Observed Delay (ms)
5	221	17,492.5
10	711	5467.1
15	491	4595.1
20	561	4912.3

## Data Availability

Additional data will be provided upon request.

## References

[1] Chen F., Wu X., Wang Z., Qi W., Li P. (2026). A New Ant Colony Optimization-Based Dynamic Path Planning and Energy Optimization Model in Wireless Sensor Networks for Mobile Sink by Using Mixed-Integer Linear Programming. Biomimetics.

[2] Wu X., Tan L., Tang S. (2020). Optimal Energy Supplementary and Data Transmission Schedule for Energy Harvesting Transmitter with Reliable Energy Backup. IEEE Access.

[3] Cumia M., Mujica G., Portilla J. (2025). Extending battery lifespan in IoT extreme sensor networks through collaborative reinforcement learning-powered task offloading. Internet Things.

[4] Jain K.A., Jain S., Mathur G. (2024). Optimizing wireless sensor network routing with Q-learning: Enhancing energy efficiency and network longevity. Eng. Res. Express.

[5] Li C., Wu J., Zhang Z., Lv A. (2023). Energy-harvesting Q-learning secure routing algorithm with authenticated-encryption for WSN. ICT Express.

[6] Abraham R., Vadivel M. (2023). An Energy Efficient Wireless Sensor Network with Flamingo Search Algorithm Based Cluster Head Selection. Wirel. Pers. Commun..

[7] Sathyamoorthy M., Kuppusamy S., Dhanaraj R.K., Ravi V. (2021). Improved K-Means Based Q Learning Algorithm for Optimal Clustering and Node Balancing in WSN. Wirel. Pers. Commun..

[8] Kanok C., Wekin P., Kampol W. (2021). Optimal Data Transfer of SEH-WSN Node via MDP Based on Duty Cycle and Battery Energy. IEEE Access.

[9] Vijayalakshmi K., Maheshwari A., Saravanan K., Vidyasagar S., Kalyanasundaram V., Sattianadan D., Victoriia B., Narayanamoorthi R. (2025). A novel network lifetime maximization technique in WSN using energy efficient algorithms. Sci. Rep..

[10] Iftikhar A., Elmagzoub M.A., Shah A.M., Al Salem H.A., ul Hassan M., Alqahtani J., Shaikh A. (2023). Efficient Energy and Delay Reduction Model for Wireless Sensor Networks. Comput. Syst. Sci. Eng..

[11] Wang Z., Song S., Cheng S. (2025). Path planning of mobile robot based on improved double deep Q-network algorithm. Front. Neurorobot..

[12] Xu L., Xi M., Gao R., Ye Z., He Z. (2025). Dynamic path planning of UAV with least inflection point based on adaptive neighborhood A* algorithm and multi-strategy fusion. Sci. Rep..

[13] Lin S., Wang J., Huang B., Kong X., Yang H. (2025). Bio particle swarm optimization and reinforcement learning algorithm for path planning of automated guided vehicles in dynamic industrial environments. Sci. Rep..

[14] Veisi M., Naderian H., Karimi M. (2025). Optimal charging station placement of electric vehicles in the smart distribution network based on the mixed integer linear programming. Int. J. Electr. Power Energy Syst..

[15] Samouilidou M.E., Passalis N., Georgiadis G.P., Georgiadis M.C. (2025). Enhancing industrial scheduling through machine learning: A synergistic approach with predictive modeling and clustering. Comput. Chem. Eng..

[16] Ghilardi L.M.P., Casella F., Barbati D., Palazzo R., Martelli E. (2025). A detailed MILP model and an ad hoc decomposition algorithm for the operational optimization of gas transport networks. Comput. Chem. Eng..

[17] Zhang Z., Li Y., Yoon S.W., Won D. (2025). Stage-based neural network for reflow profile prediction and reflow recipe optimization for quality and energy-saving. Int. J. Adv. Manuf. Technol..

[18] Ding J. (2024). The research on the solutions of the energy consumption problem in WSN in medical field. Appl. Comput. Eng..

[19] Xie W., Bai X. Research on Data Collection Mechanism of Wireless Sensor Network Based on UAV. Proceedings of the 2021 2nd International Conference on Computing, Networks and Internet of Things.

[20] Ramachandra S., Baskar M. (2025). Real-time multi-level trust-based secure routing for improved QoS in WSN using blockchain. Results Eng..

[21] Raghini M., Durairaj S., Sasikala S. (2025). Hybrid key management WSN protocol to enhance network performance using ML techniques for IoT application in cloud environment. Peer-to-Peer Netw. Appl..

[22] Kumar S.P., Nagendranath M.V.S.S., Alsamri J., Ebad S.A. (2025). Enhanced Deep Learning-Based Optimization Model for the Coverage Optimization in Wireless Sensor Networks. Int. J. Comput. Intell. Syst..

[23] Georgiadis P.G., Dimitriadis N.C., Passalis N., Georgiadis M.C. (2025). A hybrid ML-MILP framework for the optimal integration of photovoltaic and battery systems in manufacturing industries. Comput. Chem. Eng..

[24] Shabanian-Poodeh M., Ahmadi-darab F., Shafie-khah M., Kia R. (2025). Hybrid risk-based transactive energy strategy for 100% renewable-powered energy systems considering pump stations of wastewater treatment plants: A decentralized MILP model. Energy.

[25] Kassab F.A., Celik B., Locment F., Sechilariu M., Liaquat S., Hansen T.M. (2025). Microgrid sizing with EV flexibility: Cascaded MILP and embedded APSO-MILP approaches. Appl. Energy.

[26] Zelaschi A., Pliotti L., Betti G., Tonno G., Sgrò D., Martelli E. (2025). An effective MILP model for the optimal design of microgrids with high-reliability requirements. Appl. Energy.

[27] Izanlo A., Sheikholeslami A., Gholamian S.A., Kazemi M.V., Hosseini S.N. (2024). A combination of MILP and game theory methods for P2P energy trading by considering network constraints. Appl. Energy.

[28] Castelli A.F., Pilotti L., Monchieri A., Martelli E. (2024). Optimal design of aggregated energy systems with (N-1) reliability: MILP models and decomposition algorithms. Appl. Energy.

[29] Sousa M.S., da Fonseca Neto J.V. (2025). On the Minimum Quantity of Mobile Sensor Nodes for Full Coverage in Hybrid WSN. Sensors.

[30] Tossa F., Faga Y., Abdou W., Ezin E.C., Gouton P. (2025). Wireless Sensor Network Deployment: Architecture, Objectives, and Methodologies. Sensors.

[31] Suganyadevi K., Nandalal V. (2024). Swarm Intelligence-Inspired Meta-Heuristics Hybrid Optimization for Multi-Constraint Routing in Vehicular Adhoc Networks. IETE J. Res..

[32] Fakhreldin M., Jeribi F., Adam Y.A., Khawaji A., Elhadi H., Hamdan A.A. Bio-Inspired Swarm Intelligence for Optimized Energy-Efficient Computing in Smart IoT Ecosystems. Proceedings of the 2025 IEEE 9th International Conference on Software Engineering & Computer Systems (ICSECS).

